# Current Therapeutic Approach to Atrial Fibrillation in Patients with Congenital Hemophilia

**DOI:** 10.3390/jpm12040519

**Published:** 2022-03-23

**Authors:** Minerva Codruta Badescu, Oana Viola Badulescu, Lacramioara Ionela Butnariu, Mariana Floria, Manuela Ciocoiu, Irina-Iuliana Costache, Diana Popescu, Ioana Bratoiu, Oana Nicoleta Buliga-Finis, Ciprian Rezus

**Affiliations:** 1 Department of Internal Medicine, “Grigore T. Popa” University of Medicine and Pharmacy, 700115 Iasi, Romania; minerva.badescu@umfiasi.ro (M.C.B.); irina.costache@umfiasi.ro (I.-I.C.); dr.popescu.diana@gmail.com (D.P.); oana_finish@yahoo.com (O.N.B.-F.); ciprian.rezus@umfiasi.ro (C.R.); 2 III Internal Medicine Clinic, “St. Spiridon” County Emergency Clinical Hospital, 700111 Iasi, Romania; 3 Department of Pathophysiology, “Grigore T. Popa” University of Medicine and Pharmacy, 700115 Iasi, Romania; oana.badulescu@umfiasi.ro (O.V.B.); manuela.ciocoiu@umfiasi.ro (M.C.); 4 Hematology Clinic, “St. Spiridon” County Emergency Clinical Hospital, 700111 Iasi, Romania; 5 Department of Mother and Child Medicine, “Grigore T. Popa” University of Medicine and Pharmacy, 700115 Iasi, Romania; 6 Internal Medicine Clinic, “Dr. Iacob Czihac” Emergency Military Clinical Hospital Iasi, 700483 Iasi, Romania; 7 Cardiology Clinic, “St. Spiridon” County Emergency Clinical Hospital, 700111 Iasi, Romania; 8 Department of Rheumatology and Physiotherapy, “Grigore T. Popa” University of Medicine and Pharmacy, 700115 Iasi, Romania; ioana.bratoiu@umfiasi.ro

**Keywords:** atrial fibrillation, anticoagulant, catheter ablation, left atrial appendage occlusion, cardiovascular disease, hemophilia

## Abstract

Cardiovascular disease in hemophiliacs has an increasing prevalence due to the aging of this population. Hemophiliacs are perceived as having a high bleeding risk due to the coagulation factor VIII/IX deficiency, but it is currently acknowledged that they also have an important ischemic risk. The treatment of atrial fibrillation (AF) is particularly challenging since it usually requires anticoagulant treatment. The CHA_2_DS_2_-VASc score is used to estimate the risk of stroke and peripheral embolism, and along with the severity of hemophilia, guide the therapeutic strategy. Our work provides the most complete, structured, and updated analysis of the current therapeutic approach of AF in hemophiliacs, emphasizing that there is a growing interest in therapeutic strategies that allow for short-term anticoagulant therapy. Catheter ablation and left atrial appendage occlusion have proven to be efficient and safe procedures in hemophiliacs, if appropriate replacement therapy can be provided.

## 1. Introduction

Hemophilia is a rare genetic disorder. It is caused by inherited X-linked mutations in the genes encoding coagulation factor VIII (hemophilia A) or factor IX (hemophilia B). It is characterized by a lifelong increased tendency to bleed, which is generally proportional to the degree of coagulation factor deficiency. Of the two types, hemophilia A (HA) is six times more prevalent than hemophilia B (HB) [1]. While women are carriers, men clinically express the disease.

Due to advances in treating hemophilia, the life expectancy of hemophiliacs has currently increased, approaching that of the general male population [2,3,4,5,6]. Once uncommon, age-related diseases have emerged in hemophiliacs and the number of cases is growing. Among the most frequent non-hemophilia-related medical comorbidities are cancer and cardiovascular disease, both associated with old age. Their diagnosis and treatment are extremely challenging because it often requires procedures and drugs that may worsen the already deficient hemostasis. The treatment of cardiovascular diseases in patients from the general population usually includes antithrombotic therapy, either anticoagulant, antiplatelet agents, or both. Evidence-based guidelines for the management of cardiovascular diseases in hemophiliacs are lacking and the antithrombotic treatment is guided by the consensus of experts based on the international guidelines dedicated to non-hemophilia patients [1,7,8,9].

Atrial fibrillation (AF) is the most common sustained cardiac arrhythmia in the general adult population. The prevalence is 2–4% and rises due to the increase in the number of elderly people worldwide and the intensification of the effort to diagnose AF [10,11,12]. Observational studies note similar rates of AF in patients with hemophilia compared to the general population [13,14]. Since its treatment usually involves anticoagulant therapy and interventional techniques, AF poses unique therapeutic challenges in hemophiliacs because these patients have a bleeding-prone hemostatic balance. Our work provides an in-depth analysis of the modern therapeutic approach to AF in hemophiliacs, taking into account the new advances in the field. In order to extract the necessary data, we performed an extensive search in the Web of Science. The keywords “atrial fibrillation” and (“hemophilia” or “haemophilia”) were searched in the titles and abstracts of the articles. As this review aimed to provide information on the current treatment of atrial fibrillation, the search was restricted to the last 10 years. We manually screened the titles and abstracts of all 138 articles retrieved from the automatic search, removing duplicates and articles in languages other than English.

## 2. AF Epidemiology and Risk Factors

Age and many of the comorbidities associated with aging are important risk factors for AF [12,15,16,17,18,19,20,21,22,23,24,25,26,27]. Current evidence shows that hypertension, diabetes mellitus, dyslipidemia, obesity, and smoking are very common among hemophiliacs [28,29,30,31,32]. Moreover, of these cardiovascular risk factors, hypertension is more prevalent in hemophiliacs than in the general male population [29,30,31,32,33]. The clustering of cardiovascular risk factors is also present, with more than a third of hemophiliacs having metabolic syndrome [33]. The unfavorable cardiovascular risk profile sets in earlier in life in hemophiliacs than in the general population [29,31,34] and the cardiovascular risk factor burden is present, irrespective of hemophilia severity [32,35].

In men, the risk of AF increases steeply after the age of 50 [36]. Thus, with the prolongation of the lifespan of hemophiliacs and the accumulation of risk factors for AF, the number of patients with this arrhythmia is expected to increase. In the largest European cohort of hemophiliacs analyzed to date, the prevalence of AF was 0.84% [13]. A low value of 0.2% was found in patients younger than 60 years of age and seventeen times higher (3.4%) in those over 60 years of age, similar to that of the general population [9]. While there was a very important age-dependent upward trend, the prevalence of AF was inversely correlated with the severity of hemophilia. Other European studies reported a prevalence of 2.2–2.4% [37,38]. The lowest prevalence of AF was found in Asian hemophiliacs (0.28%) [30], and the highest in two North American cohorts, 5.4% in young hemophiliacs [39] and 7.5% in those aged between 54 and 73 [32]. This highlights the negative impact of aging on the prevalence of AF.

## 3. Assessment of the Ischemic Risk

Blood stasis in the left atrium and impaired contractility of the left atrial appendage are the main contributors to cardioembolism [40]. It is considered that 20–30% of all ischemic strokes and 10% of cryptogenic strokes are the consequence of AF [12]. Generally, AF multiplies the risk of stroke by five [41]. This risk is not homogeneous in the general population, but is dependent on comorbidities and ranges between 1% and 20% [42,43]. Moreover, strokes associated with AF are usually severe, highly recurrent, often fatal, or with permanent disability [12].

The risk of AF-related stroke is influenced by additional factors, therefore, risk scores have been developed to stratify patients and guide the anticoagulant treatment [44,45,46]. The CHADS_2_ score was originally used [47], but the CHA_2_DS_2_-VASc score outperformed the CHADS_2_ score and simplified the anticoagulation decision-making as it could identify individuals with a truly low thromboembolic risk that do not require anticoagulant treatment (0 in men and 1 in women) [48,49].

Data on the risk of ischemic stroke in hemophiliacs compared to men in the general population are scarce. Still, the rate of ischemic stroke is expected to be low [50]. A retrospective study showed that the prevalence of ischemic stroke was lower in hemophiliacs with severe disease (0%) than in the general male population (1.5%), but this difference was not statistically significant in hemophiliacs with mild-moderate disease [37]. Of four patients with ischemic stroke, the event was correlated with the presence of AF in one.

Few data are available on the risk of ischemic stroke and systemic embolism in hemophiliacs with AF. CHADS_2_ and subsequently CHA_2_DS_2_-VASc scores were used to estimate this risk, but none have been prospectively validated in a population of hemophiliacs thus far. The CHA_2_DS_2_-VASc score was calculated in 33 patients with AF from a large pan-European cohort of almost 4000 hemophiliacs [13]. The score ranged from 0 to 4, driven principally by age and hypertension. The score’s mean value was 1.3. Of the 33 hemophiliacs with AF, 16 (48%) had hypertension, three (9%) peripheral vascular disease, three (9%) diabetes, and two (6%) had previous stroke. None had congestive heart failure. Eleven patients (33%) were between 65 and 74 years and three (9%) were 75 years or older. There were ten hemophiliacs with a CHA_2_DS_2_-VASc score of 0. Based on the CHA_2_DS_2_-VASc score, in hemophiliacs with AF in this cohort, the risk of stroke was low [13].

The French registry included 68 hemophiliacs requiring antithrombotic treatment for acute coronary syndromes (ACS)/coronary artery disease (CAD), AF, or both [51]. Seventeen patients only had AF and one patient had both AF and CAD. The CHA_2_DS_2_-VASc score ranged from one to seven. Sixteen patients had a CHA_2_DS_2_-VASc score ≥2. The score’s mean value was three, much higher than previously reported [13]. This difference was not determined by the age of the enrolled patients, but by the load of comorbidities.

Data also came from a short series of cases and case reports. In seven HA patients, the CHA_2_DS_2_-VASc score ranged between one and six, with a mean value of three, where age and hypertension were the most important contributors [52]. The patient with severe hemophilia had the highest CHA_2_DS_2_-VASc score mainly due to advanced age, previous stroke, and myocardial infarction. Cheung et al. reported the case of a HA patient with mild disease who also had a score of six due to age, left ventricular dysfunction, hypertension, history of stroke, and atherosclerotic carotid stenosis [53]. A very high CHA_2_DS_2_-VASc score of seven was reported in a 79-year-old patient with mild HA, based on age, chronic heart failure, hypertension, transient ischemic attack, and coronary artery disease [54].

Although the value of the CHA_2_DS_2_-VASc score varies widely in this population, it has become evident that due to the multiple risk factors that accumulate with age, hemophiliacs with AF have an increased risk of ischemic stroke and systemic embolism, regardless of the severity of the disease. Therefore, stroke prevention measures should be discussed with the patient and implemented by a cardiologist–hematologist team.

The first attempt to incorporate a stroke risk score in anticoagulation decision-making was based on the CHADS_2_ score [55]. Currently, the CHA_2_DS_2_-VASc score is endorsed by the ADVANCE (age-related developments and comorbidities in hemophilia) Working Group [8]. The proposed algorithm incorporates both the CHA_2_DS_2_-VASc score and the severity of hemophilia. Still, caution is advised as the absolute risk of stroke may be overestimated because the CHA_2_DS_2_-VASc score includes parameters that are either irrelevant—female sex—or rarely relevant— peripheral arterial disease and very old age—for the hemophilia population. It has been proposed that only hemophiliacs with a CHA_2_DS_2_-VASc score ≥3 should be considered with a high risk of stroke [50]. Considering the severity of hemophilia and the risk of bleeding, other researchers have considered that a CHA_2_DS_2_-VASc score ≥2 should be used to start anticoagulation in patients with factor level ≥20% and a higher threshold—CHA_2_DS_2_-VASc score ≥4—was suggested in hemophiliacs with a factor level <20% [8,9].

## 4. Assessment of the Bleeding Risk

The HAS-BLED score is used in the general AF population for estimating the basal bleeding risk before and during the anticoagulant treatment [56]. A HAS-BLED score ≥3 indicates a high risk of bleeding, but this value should not be used as an absolute parameter for withholding or withdrawing anticoagulation. This score must be seen as a tool to reduce the bleeding risk by identifying those risk factors that can be avoided or reversed.

The reliability of this score in hemophiliacs has long been questioned because two major determinants of the risk of bleeding in hemophiliacs—the severity of the disease and the presence of inhibitors—are not directly assessed by this score.

The frequency of bleedings depends on the severity of hemophilia. The highest risk is in patients with a factor level <1% of normal, who may bleed spontaneously or after minimal trauma [57,58]. Hemophiliacs have a particular hemorrhagic profile. Bleedings are usually recurrent and mainly affect the joints and muscles. Although it is a rare complication, intracranial hemorrhage (ICH) is the most feared because it is life-threatening. Its incidence is higher in hemophiliacs than in the general population, especially in those with severe disease [59,60]. The implementation of modern therapies has led to a major reduction in ICH incidence so that currently, the patients on prophylactic treatment no longer develop ICH [59,60,61,62]. Moreover, hypertension has long been associated with ICH [63]. Since hypertension is very prevalent in hemophiliacs, its diagnosis and intensive treatment should be promptly implemented to reduce the ICH risk in the entire hemophiliac population.

Hemophilia patients without inhibitors typically have a predictable response to clotting factor replacement, and bleeding can be reliably prevented or treated. Those with baseline factor levels >5% and those with severe hemophilia under clotting factor prophylaxis have the lowest risk of bleeding [7]. Patients with inhibitors have a much less predictable hemostatic response to bypassing agents. The problem with developing neutralizing anti-FVIII antibodies is of great importance as it occurs in 25–33% of HA patients [64,65]. Studies have found that 10–20% of bleedings in hemophiliacs with inhibitors are either unresponsive or only partially responsive to bypassing agents [66]. Therefore, the risk of uncontrolled bleeding almost always outweighs the benefit of anticoagulation.

When reported, often in a small series of cases and case reports, the HAS-BLED score was frequently three. In seven HA patients, the HAS-BLED score ranged between one and three, with a mean value of three. Higher values of the score such as five or six have also been reported [54,67].

Until 2021, when the results of the French registry were published, there was a general agreement that the HAS-BLED score underestimated the bleeding risk in hemophiliacs and was considered not suitable for use in this population [50,68]. The French registry provided evidence to the contrary. The HAS-BLED score of the 18 hemophiliacs with AF ranged from zero to four, with a mean value of two [51]. A HAS-BLED score ≥3 was associated with increased bleeding risk. In the two years of follow-up under antithrombotic treatment, five out of eight patients with a HAS-BLED score ≥3 reported bleeding episodes, while no hemorrhagic events were reported in any of the 10 patients with a HAS-BLED score <3. Of note, none of the hemophiliacs had inhibitors. The median coagulation factor level and the proportion of patients on prophylaxis were similar between the two groups. This was the first report to show that the HAS-BLED score is applicable to hemophiliacs.

The type, intensity, and duration of antithrombotic therapy are determined, on one hand, by the ischemic risk, and on the other hand, by the risk of bleeding and need for replacement therapy, which must ensure a level of coagulation factor that allows for safe antithrombotic treatment. Intensive replacement therapy as required by anticoagulants is unlikely to be sustainable in the long-term. In this context, finding alternative therapeutic solutions has become a priority. Of particular interest are the catheter ablation techniques and the devices for closing the left atrial appendage.

## 5. Rhythm or Rate Control in AF

When choosing between rhythm and rate control, it should be considered that over time, AF becomes less responsive to treatment or is irreversible [69] and that AF progression leads to an increased risk of ischemic stroke or systemic embolism, a high risk of hospitalization for heart failure [70] and a decrease in the quality of life [71]. The current guideline highlights the fact that the primary indication for rhythm control is to reduce the symptom burden related to the arrhythmia and to improve the quality of life, especially when factors favoring rhythm control are prevailing [12]. While no major differences were observed in cardiovascular mortality or stroke rate [72], the rhythm control strategy improved the left ventricular function and quality of life in patients with heart failure [73].

The drugs used for rate or rhythm control and the antiarrhythmic selection algorithm in hemophiliacs mirror the guidelines for AF management in the general population [12]. One option to achieve rhythm control is through cardioversion and the currently available data in hemophiliacs reflect a preference for the pharmacologic approach [74,75,76,77,78]. The anticoagulant therapy should be started as soon as possible before cardioversion, and concomitant coagulation factor replacement therapy should be administered when necessary. The algorithm that includes transesophageal echocardiography (TEE) should be considered first—when available—because TEE can rule out atrial thrombi and the 3-week period of anticoagulation prior to procedure is avoided [12]. Of note, successful cardioversion does not imply that long-term anticoagulant treatment is no longer necessary. The stroke occurrence in high-risk patients is the same, regardless of the rhythm or rate control strategy adopted [79], therefore, the decision regarding long-term oral anticoagulant treatment should be driven by the presence of the stroke risk factors [12]. Thus, other therapeutic options should be considered in hemophiliacs with a CHA_2_DS_2_-VASc score of one or higher in whom long-term anticoagulant therapy treatment is not feasible.

Patients with AF require both antiarrhythmic and anticoagulant treatment, therefore, the guidelines provide specific recommendations to help in the selection of the appropriate drug and dose [80]. Recent data on clinical implications of drug–drug interactions show that in patients over 66 years old on anticoagulant treatment with a NOAC —apixaban, dabigatran, rivaroxaban—the bleeding risk was not increased by the concomitant use of amiodarone, diltiazem, and verapamil [81]. If dronedarone is used, it should be noted that it increases the frequency of gastrointestinal bleedings when associated with dabigatran and rivaroxaban and the risk of overall bleeding in patients receiving rivaroxaban [82]. Thus, when selecting AF treatment for hemophiliacs, it should be considered that the underlying hemorrhagic risk will be increased by the administration of the antithrombotic therapy and that some antiarrhythmic drugs may further potentiate this risk.

Due to the increased bleeding risk of hemophiliacs, there is a permanent pursuit for the use of the minimum effective dose of anticoagulant. The increasing availability of thrombin generation assays could facilitate this approach. While conventional coagulation tests provide only partial information on hemostasis, the global coagulation tests evaluate the functionality of all its components. By assessing the dynamics of clot formation, clot resistance, and stability, global coagulation tests reflect the interaction between procoagulants, natural anticoagulants, platelets, and the fibrinolytic system. Guided by a thrombin generation assay, a reduced dose of low molecular weight heparin was safely administered to a HA patient before cardioversion [68].

## 6. Surgical and Catheter Ablation of AF

AF catheter ablation for pulmonary vein isolation (PVI) is an efficient and safe rhythm control strategy. It represents a first-line therapy in selected patients with symptomatic AF and it is also recommended after antiarrhythmic drug therapy failure [12,83,84]. The interest in catheter ablation is growing because this therapy is perceived as a way to avoid long-term treatment with oral anticoagulants. It was hypothesized that after successful ablation, the risk of thromboembolic events in patients with AF will be the same as in patients without AF [85,86,87], thus the anticoagulant treatment could be stopped. However, discontinuation of oral anticoagulant therapy is associated with a low risk of stroke only in patients with a low CHA_2_DS_2_-VASc score [84]. Patients with a previous stroke continue to have a high risk of thromboembolic events despite the successful procedure [88].

Anticoagulant treatment is necessary during the procedure to prevent thrombus formation on sheaths and catheters and at the sites of ablation. The standard protocol using unfractionated heparin is also implemented in hemophiliacs given that during the procedure, the coagulation is restored to normal by the factor replacement therapy [78,84]. Most centers adopt the 300–350 s interval [78], but a slightly lower activated clotting time (ACT) target has been used [77]. There is evidence that even an ACT target range of 225–250 s is safe and effective in specified settings [89,90].

AF ablation is followed by a period of high embolic risk due to atrial stunning, tissue damage caused by the procedure, and possible early recurrence of arrhythmia [77]. Therefore, the anticoagulant treatment is recommended for at least two months after the procedure [12]. Because the predictive value of the CHA_2_DS_2_-VASc score is maintained after AF ablation regardless of the arrhythmic outcome [88], it is recommended that the decision to stop or continue anticoagulation beyond two months be made based on the patient’s risk profile and not on the result of the ablation procedure [12,84].

Cardiac ablation includes the catheter, surgical, and hybrid surgical–catheter ablation. Data on protocol and outcome of the catheter ablation procedure for AF treatment in hemophiliacs are very recent (Table 1). Cryoablation was successfully performed in a HA patient with symptomatic AF that could not be amended through multiple pharmacological attempts [77]. Anticoagulant and replacement factor therapy were used during the PVI procedure, but no long-term antithrombotic therapy was recommended due to the patient’s CHA_2_DS_2_-VASc score of 0.

A total of seven PVI procedures for symptomatic AF—with an average duration of five years—were performed in five HA patients [78]. Three patients remained in sinus rhythm during follow-up, while in two cases, the procedure was repeated at nine and 16 months after the first PVI due to AF recurrence. The success rate of PVI was comparable to that reported in the general population [91] and the complications were only hematoma at the femoral vein puncture site. Procedures were performed under anticoagulant treatment and replacement factor therapy, aiming at a peak coagulation factor level of 80–100% during ablation and in the following 24 h. On the first day after the procedure, the trough level of the coagulation factor was kept above 50%. For at least four weeks after the procedure, patients received either dabigatran 110 mg bid or VKA and bridging with low-molecular-weight heparin (LMWH) until INR >2. A coagulation factor level of at least 20% was considered safe during the anticoagulant treatment.

Surgical AF ablation performed concomitantly with cardiac surgery is an opportunity to add supplementary benefits [92]. A 50-year-old HA patient with mild disease underwent minimally invasive mitral valve repair for valve leaflets prolapse with severe mitral regurgitation. Exclusion of the left atrial appendage and left and right atrial cryoablation for concurrent AF was also performed [93]. His CHA_2_DS_2_-VASc score was one. At a 2-year follow-up, the patient had sinus rhythm without AF paroxysms. No antithrombotic was recommended after surgery.

Severe complications after catheter ablation occur in 5–7% of patients in the general population, of which 2–3% are life-threatening. Stroke (<1%) and pericardial tamponade (1–2%) are the most severe. Clinical relevant hematoma at the site of venous puncture occurs in 0.9% of patients in the general population. Periprocedural inguinal bleeding has been reported in hemophiliacs, in two cases with a significant decrease in the hemoglobin level [78]. No fatalities have been reported in hemophiliacs to date.

## 7. Percutaneous Left Atrial Appendage Occlusion

As more than 90% of emboli originate in the left atrial appendage (LAA) [99], percutaneous left atrial appendage occlusion (LAAO) arose from the need to provide protection from ischemic stroke and systemic embolism in AF patients for whom long-term anticoagulant treatment is contraindicated. Data provided by several studies confirmed that LAAO is a feasible, effective and safe alternative to oral anticoagulant treatment [100]. LAAO has comparable efficacy with VKA at a lower rate of major bleedings, particularly hemorrhagic stroke [101,102]. This strategy provides a 90% reduction in the rate of hemorrhagic stroke [103]. In high-risk AF patients, LAAO has comparable efficacy with NOAC in stroke prevention and a similar or better safety profile [104,105]. To prevent device-related thrombosis after implantation, antithrombotic treatment is required until the complete endothelialization of the device surface. In patients without contraindications to oral anticoagulation, the postprocedural antithrombotic therapy consists of 45 days of VKA/NOAC and aspirin, followed by six months of dual antiplatelet therapy (DAPT) with aspirin and clopidogrel and then lifelong single antiplatelet therapy (SAPT) with aspirin [100].

Patients who have had bleeding complications or who have contraindications to long-term oral anticoagulant treatment will receive DAPT with aspirin and clopidogrel for 1–6 months, followed by lifelong SAPT with aspirin [100]. In naïve patients, loading doses of aspirin and clopidogrel are indicated. Patients at extremely high risk of bleeding should receive SAPT for at least 2–4 weeks. If no antithrombotic drug can be administered, epicardial LAA occlusion or thoracoscopic LAA clipping should be considered instead of LAA occluder implantation [100]. Early data from an ongoing study suggest that reduced doses of rivaroxaban (10 mg od and 15 mg od) may be an alternative to postprocedural DAPT [106].

Watchman, Amplatzer Cardiac Plug, and Amulet devices are the most widely used (Table 1). The Amplatzer device family is less thrombogenic than the Watchman device [107], therefore, postprocedural anticoagulation is never necessary [12]. The patients receiving Amplatzer Amulet devices did not have a higher risk of device-related thrombosis while on antiplatelets, especially those on SAPT [108]. In patients at high risk of stroke and contraindication to oral anticoagulant treatment, the implantation of Watchman device followed by DAPT was also an effective strategy [109]. The first case of LAAO in a hemophiliac with a high risk of stroke due to AF was published ten years ago [53]. Since then, many successful implantations of Amplatzer Cardiac Plug and Amplatzer Amulet devices have been reported [53,67,95,96]. Patients had high ischemic and bleeding risks. Their CHA_2_DS_2_-VASc score ranged from three to six and the HAS-BLED score from three to six. The postprocedural antithrombotic treatment consisted of antiplatelet agents.

Successful use of the Watchman device was also reported in hemophiliacs with AF [54,98]. Postprocedural antithrombotic therapy varied widely, from anticoagulation to no treatment. In a 60-year-old HB patient with mild disease, AF catheter ablation and placement of the Watchman device were performed concomitantly in order to minimize the patient’s exposure to the anticoagulant [94]. After the procedure, the patients received only one month of VKA treatment. Still, the outcome was favorable at a 6-month follow-up.

A combined therapy consisting of LAA closure and cardioversion was successfully performed in a 69-year-old patient with moderate HB while on apixaban 2.5 mg bid and prophylaxis with eftrenonacog alfa [97]. After the procedure, he received one-month apixaban, three months DAPT, and long-term clopidogrel with favorable outcomes.

The largest series of cases of percutaneous LAAO published to date included seven patients with HA [52]. Three patients received the Amplatzer Amulet device and the rest the Watchman device. Before the procedure, two patients were on NOAC, one on LMWH, and one on DAPT and LMWH. After the device implantation, only antiplatelets were recommended, mainly SAPT with aspirin. In two cases, short-term DAPT was used.

Procedural risks associated with LAAO are cardiac tamponade (2–4%), stroke (1–2%), and inguinal hematoma (1%). Device embolization is extremely rare (<1%), but may require emergency cardiac surgery [103,110]. Most complications in hemophiliacs were minor or significant bleedings at the site of venous puncture. In one case, periprocedural arterial puncture occurred and in two cases, pericardial effusion without tamponade was diagnosed [52,67].

Although periprocedural therapy varies between treating centers, prophylactic replacement of the coagulation factor is always necessary, as a very high factor level of ≥80% must be achieved during the procedure [52,53,54]. This level of coagulation factor is the same as that recommended for major surgery [57]. The percutaneous LAAO procedure is minimally invasive and does not require such intense replacement therapy, but the high level is considered necessary due to the risk of intracardiac lesions and the possible conversion to thoracic surgery. Moreover, full heparinization is used during the procedure [52,54,94]. In the first three days after the procedure, a trough level of the coagulation factor of 80% was frequently used [52,53].

## 8. Long-Term Antithrombotic Treatment

Current guidelines do not support the use of antiplatelet therapy alone for stroke prevention in AF patients in the general population [12]. It was proven that aspirin alone has limited or no protective effect against ischemic stroke [111,112]. Adding clopidogrel to aspirin reduced the stroke rate compared to aspirin alone, but major bleedings significantly increased [113]. Moreover, DAPT with aspirin and clopidogrel did not offer better protection from stroke than VKA at a similar rate of major bleedings [114].

AF patients requiring antithrombotic treatment receive an anticoagulant. VKA is more effective than aspirin in preventing ischemic stroke, with similar rates of major bleedings even in those older than 75 years [115]. Four NOACs—apixaban, dabigatran, edoxaban, and rivaroxaban—are currently indicated for stroke prevention in AF patients. A meta-analysis of their pivotal trials showed that NOACs reduced by 19% the risk of stroke or systemic embolism, with 51% in the risk of hemorrhagic stroke, and with 10% all-cause mortality comparative to VKA [116]. There was also a 14% reduction in the risk of major bleedings with NOACs, most coming from a 52% reduction in intracranial hemorrhages. Still, gastrointestinal bleedings increased by 25% with NOACs compared to warfarin. Of note, fatal bleedings are halved by using a NOAC versus VKA [117]. Due to the more favorable benefit–risk ratio, NOACs are preferred over VKAs in patients with AF eligible for a NOAC—without prosthetic mechanical heart valves or moderate-severe mitral stenosis [12].

In men with AF in the general population, the anticoagulant treatment should be considered if the CHA_2_DS_2_-VASc score is ≥1 [12]. Since there is no evidence that hemophiliacs are protected from cardioembolism by their underlying coagulation defect [57], the antithrombotic treatment should be considered in the presence of AF. Table 2 provides a practical algorithm for the antithrombotic treatment.

The most recent therapeutic algorithm was proposed by Schutgens et al. and is based on both the CHA_2_DS_2_-VASc score and the severity of hemophilia [8]. Among hemophiliacs with baseline FVIII/IX level ≥20%, the anticoagulant treatment is recommended only if the CHA_2_DS_2_-VASc score is ≥2. All oral anticoagulants are allowed, but the use of NOACs over VKA is encouraged because of the superior safety profile of NOACs to that of VKA, particularly regarding intracranial bleeding [116]. NOACs are preferred, especially in patients with HB because VKA reduces plasma FIX levels and increases the severity of hemophilia and consequently the risk of intracranial hemorrhage. While on VKA, these patients will need even more intensive replacement therapy, which is burdensome and may increase the frequency of inhibitor development. The high frequency of intravenous coagulation factor infusion and the related costs make long-term VKA use prohibitive [94].

The plasma FVIII/IX level considered safe for oral anticoagulation is ≥30% [7,55]. Thus, only a few patients will benefit from anticoagulant treatment without requiring replacement therapy, namely the patients with very mild hemophilia and native factor activity level above this threshold. For hemophiliacs with more severe disease, this high threshold is difficult to maintain in the long-term because it requires very frequent administration of replacement products, often every 1–2 days [51]. Several studies have reported the safe use of anticoagulant treatment in hemophiliacs with FVIII/IX levels ≥20% [118,119] including after catheter ablation [78]. Therefore, it has been proposed to lower the threshold from 30% to 20% [8].

Dabigatran is a direct thrombin inhibitor. In its pivotal trial, the dose of 150 mg bid reduced the risk of stroke by 36% compared to VKA at a similar rate of major bleedings. The dose of 110 mg bid was associated with a similar risk of stroke and a lower rate of major bleedings [120]. Of note, both regimes were associated with lower rates of hemorrhagic stroke than VKA, but the 150 mg bid dose increased the incidence of gastrointestinal bleeding by 50%. Dabigatran was successfully administered in hemophiliacs with AF eligible for anticoagulant treatment [8,121]. The 110 mg bid regimen was used due to its similar efficacy to VKA at a lower risk of bleeding.

Apixaban is a direct inhibitor of FXa, more efficient and safe than VKA in preventing stroke or systemic embolism in patients with AF eligible for a NOAC [122]. A recent real-world data analysis evaluated the efficacy and safety of apixaban according to its dose [123]. The rates of stroke/systemic embolism were reduced by 30% with the 5 mg bid regimen and by 37% with the 2.5 mg bid regimen. The rates of major bleeding decreased by 41% for each of the two regimes. To date, the successful use of low-dose apixaban has been reported in hemophiliacs with AF [52,97].

Rivaroxaban is a direct inhibitor of FXa, non-inferior to VKA in preventing stroke and systemic embolism in patients with AF eligible for a NOAC and with similar overall safety [124]. In the pivotal trial, the intracranial bleedings were less frequent with rivaroxaban than VKA, but the number of gastrointestinal bleedings increased. A recent real-world dose-based data analysis of rivaroxaban showed that the 20 mg od regimen reduced the risk of stroke and systemic embolism and was associated with fewer major bleedings compared to VKA. The 15 mg od regimen was as effective and safer than VKA [125]. In Asians with AF, the efficacy and safety of the 10 mg dose were also assessed [126]. Data from studies conducted on the Asian population showed that rivaroxaban was associated with a lower risk of stroke or systemic embolism than warfarin, regardless of dose [127]. In patients with mild hemophilia, the 10 mg od regimen was used [52].

Edoxaban is a direct inhibitor of FXa non-inferior to VKA in preventing stroke or systemic embolism in patients with AF eligible for a NOAC, with significantly lower rates of major bleeding than VKA for both 60 mg od and 30 mg od regimen [128]. The 60 mg od regimen was slightly more efficient than VKA. The rates of life-threatening bleeding, intracranial bleeding, and major bleeding plus clinically relevant non-major bleeding were favorable to edoxaban for both regimes, except for gastrointestinal bleedings that were higher with 60 mg od edoxaban than with VKA and lower with 30 mg od edoxaban than with VKA.

Thus, the preference for NOACs in hemophiliacs with AF is justified as they provide protection against ischemic stroke at least as well as VKA, but with a lower hemorrhagic risk including reduction in intracranial bleeding.

Hemophiliacs with baseline FVIII/IX level 1–19% and patients with the severe disease under FVIII/IX prophylaxis are candidates for catheter ablation or antiplatelet therapy with aspirin only if the CHA_2_DS_2_-VASc score ≥4 [8]. As VKA doubles the risk of intracranial hemorrhage compared to aspirin [129], it is considered unsuitable in hemophiliacs with basal FVIII/IX level <20% [8]. The plasma FVIII/IX level considered safe for SAPT is ≥5–10% [1,7,55].

Of note, apixaban is the only NOAC that has been compared in a large randomized clinical trial with aspirin in AF patients considered unsuitable to receive a VKA [130]. The rate of stroke/systemic embolism was more than halved, while the risk of major bleeding or intracranial hemorrhage did not significantly increase. Thus, the administration of apixaban could be a solution for those hemophiliacs receiving aspirin instead of VKA because their risk of bleeding is considered too high or intensive prophylactic replacement therapy is not feasible [75].

The oral anticoagulant treatment is not recommended in hemophiliacs with severe disease without prophylaxis because the thrombin generation is comparable to that in patients with a therapeutic INR [131]. In hemophilia patients with inhibitors, the antithrombotic therapy is generally contraindicated due to the less predictable hemostatic response to bypassing agents [66].

## 9. Discussion

Modern replacement therapies of the deficient coagulation factor prolong the lifespan of hemophiliacs, enabling the occurrence of diseases associated with old age. Atherosclerotic cardiovascular disease challenges most physicians’ ability to provide adequate care because it often requires antithrombotic therapy. Hemophilia is a rare disease, so there are no conditions for conducting randomized clinical trials. Therefore, the therapeutic approach to cardiovascular diseases in hemophiliacs including AF is based on case reports, observational studies, and the consensus of experts [1,7,8,9,55], which is unlikely to change in the near future.

The management of patients with AF relies on three pillars: avoiding stroke, better symptom management, and cardiovascular and comorbidity optimization [12]. The CHA_2_DS_2_-VASc and HAS-BLED scores are recommended for the assessment of the risk of stroke and bleeding in the general population, respectively. Stroke prevention has evolved considerably in the last decade. Anticoagulant therapy with VKA or better with NOAC is the mainstream treatment. Patients with contraindication to oral anticoagulants or at risk of bleeding under anticoagulant therapy perceived as too high to be acceptable can now be candidates for left atrial appendage occlusion/exclusion [12]. Rhythm control to reduce the AF related symptoms and to improve the quality of life can be implemented through drugs or more recently, cardiac ablation. Pulmonary vein isolation is usually performed, but surgical or thoracoscopic ablation is also available in selected cases.

Although used to guide treatment in hemophiliacs with AF [7,8], the CHA_2_DS_2_-VASc score has not been prospectively validated in this population. Still, high values of this score have been found in hemophiliacs [52,53,54,67], suggesting that the risk of stroke and systemic embolism may be higher than anticipated [13]. The exact risk of stroke/systemic embolism in hemophiliacs with AF is still unknown, but it is certain that it is present even in patients with severe hemophilia. The HAS-BLED score was rarely used in hemophiliacs to estimate the bleeding risk because it was believed that the coagulation factor deficiency itself carries a high risk of bleeding. Moreover, it has been hypothesized that this score underestimates the bleeding risk in these patients [50,68]. However, recent evidence showed that a HAS-BLED score ≥3 was associated with increased bleeding risk even in hemophiliacs [51].

The antithrombotic treatment increases the risk of bleeding in all hemophiliacs, regardless of the severity of the disease. It was found that during the anticoagulant treatment, the bleeding risk of hemophiliacs with the mild disease increased eight times compared to the controls [51]. In hemophiliacs eligible for long-term oral anticoagulants, a preference for NOAC instead of VKA is highlighted, justified by their better efficacy and safety profile. Moreover, low doses of NOACs prevent stroke equal to or more than warfarin, with less major bleedings [120,122,123,124,125,126,127,128]. According to the available data, apixaban and 10 mg rivaroxaban seem to be the most suitable to recommend to hemophiliacs presenting AF [75,127,132]. Reports of successful use of NOACs in hemophiliacs with AF have already begun to appear [8,52,97].

HB patients benefit greatly from these recommendations. While a large fraction of factor VIII in blood originates from liver sinusoidal endothelial cells [133], FIX is a vitamin K-dependent coagulation factor produced in the liver. The treatment with VKA will further reduce plasma FIX levels, increasing the severity of hemophilia and the need for substitution therapy. Moreover, FIX replacement therapy may interfere with VKA and make INR value unreliable [55]. Since the coagulation factors II, VII, and X are also vitamin K-depending coagulation factors synthesized in the liver, VKA administration has the potential to further aggravate the imbalance of the hemostasis in hemophiliacs.

The experience gained so far suggests that the anticoagulant therapy is safe for hemophiliacs under appropriate replacement therapy, so working with a hematologist is vital for therapeutic success. In general, levels ≥80% and aiming at 100% are recommended intraprocedural and up to three days thereafter [52,53,54,78], ≥20% under anticoagulant treatment and DAPT [8,9,78], and ≥5–10% under SAPT [1,7,55].

The management of AF in patients with hemophilia can be quite complex, particularly for the reduction in the risk of stroke in the long-term and in the context of invasive procedures. Since the anticoagulant treatment is considered safe at FVIII/FIX level ≥20% [8], few hemophiliacs can receive it without coagulation factor replacement therapy. Many will require frequent administrations, possibly every 1–2 days, which is burdensome in the long run. It should be noted that this type of replacement therapy is also very expensive. Avoiding the need for long-term anticoagulant therapy should be a priority, therefore, the optimal therapeutic solution must be sought with great care. Cardioversion helps to control AF-related symptoms, improves left ventricular function, and quality of life, but long-term anticoagulant therapy is often needed [12].

PVI with short-term replacement therapy during periprocedural treatment with VKA or NOAC seems to be a good option [8]. NOACs appear safer than bridging VKA with LMWH for PVI [78]. Catheter ablation proved to be effective for rhythm control and safe to perform in hemophiliacs [77,78]. Although the mandatory duration of the anticoagulant treatment is two months, it is recommended that the treatment be extended in patients with high ischemic risk [12]. Still, it must also be considered that by continuing the oral anticoagulant treatment beyond three months from the procedure, the thrombotic events will slightly decrease while the major bleedings will increase [134].

Percutaneous LAAO is another possibility for these patients with a high bleeding risk [135]. It attracts increasing interest due to its favorable antithrombotic therapy profile, namely, the possibility of using short-time DAPT or anticoagulant, then lifetime SAPT. While DAPT requires the same level of coagulation factor as the anticoagulant treatment [9], SAPT may be safely recommended in hemophiliacs with factor level ≥5–10% [1,7,55]. This is a feasible approach in hemophiliacs. Thus, advances in the treatment of hemophilia allow for the implementation of the modern therapeutic solutions of AF in hemophilic patients.

This study has several limitations. First, there are little data on the management of AF in hemophiliacs, especially on percutaneous cardiac ablation and LAA occlusion techniques, therefore the conclusions cannot be generalized. Second, studies that have reported the use of antithrombotic therapy in hemophiliacs have often included, in addition to patients with AF, those with acute coronary syndromes or elective coronary procedures (coronary artery bypass grafting and percutaneous coronary intervention with stent implantation), which made it difficult to extract the necessary information. Moreover, data on the management of AF in hemophiliacs have sometimes been reported along with those from patients with other hereditary bleeding disorders.

## 10. Conclusions

Patients with hemophilia are often perceived as having only an increased risk of bleeding. However, in the presence of AF and ischemic risk factors, they also have an increased risk of stroke and systemic embolism. The choice of appropriate therapy is greatly hampered by the lack of guidelines based on strong evidence. Although the physicians treat hemophiliacs with AF on a case-by-case basis, it should be highlighted that the treatment offered in the general population can be implemented in hemophiliacs if appropriate replacement therapy can be provided. As far as we know, our work provides the most complete, structured, and updated analysis of the current therapeutic approach of AF in hemophiliacs.

## Figures and Tables

**Table 1:** Reports on the non-pharmacological treatment of AF.

Author, Year	Patient Age, Sex	Type and Severity of Hemophilia (Baseline Factor Activity Level)	CHA_2_DS_2_-VASc Score/HAS-BLED Score	Comorbidities	Procedure/Device	Antithrombotic Treatment after the Procedure and on Long-Term	Coagulation Factor Replacement Treatment	Outcome
Lin et al., 2015 [77]	54 y, M	HA, mild (5%)	0/NR	Obstructive sleep apnea, hemarthroses of peripheral joints	Catheter ablation (PVI)	No antithrombotic treatment	FVIII level 131% before the procedure	No periprocedural complications; No complications at 2-year follow-up
van der Valk et al., 2019 [78]	70 y, NR	HA, mild (35%)	3/NR	NR	Catheter ablation (PVI) Catheter ablation (PVI) -repeated	VKA 3 mo Dabigatran 110 mg bd	FVIII level ≥80% for the procedure and for the first 24h; FVIII level ≥20% while on anticoagulant (replacement therapy was given when needed)	Groin bleeding with severe anemia (day 5 after the first procedure)
	72 y, NR	HA, severe (˂1%)	1/NR		Catheter ablation (PVI) Catheter ablation (PVI) -repeated	VKA 1 mo VKA 1 mo, SAPT with aspirin 2 mo		Groin bleeding with severe anemia (day 3 after the first procedure) Oozing during 4 h at puncture site at the second procedure
	59 y, NR	HA, mild (23%)	0/NR		Catheter ablation (PVI)	Dabigatran 110 mg bd 6 mo		No periprocedural complications
	50 y, NR	HA, severe (˂1%)	0/NR		Catheter ablation (PVI)	VKA 6 wk		No periprocedural complications
	55 y, NR	HA, mild (6%)	0/NR		Catheter ablation (PVI)	Dabigatran 110 mg bd 6 wk		No periprocedural complications
Bogachev-Prokophiev et al., 2020 [93]	50 y, M	HA, severe (˂1%)	1/NR	Both mitral valve leaflets prolapse with severe regurgitation, recurrent hemarthrosis with limited mobility in the elbow and knee joints	Left and right atrial ablation; left atrial appendage was excluded	No antithrombotic treatment	FVIII level 109% before the procedure	Moderate HF and supraventricular tachycardia during hospitalization; Class I NYHA HF at 2-year follow-up
Cheung et al., 2013 [53]	73 y, M,	HA, mild (8%)	6/NR	CABG, stroke, 90% stenosis of the right ICA from calcified plaque amended by endarterectomy, 50–70% stenosis of the left ICA, hypertension, moderate left ventricular impairment, hypercholesterolemia, hepatitis C, COPD	Amplatzer Cardiac Plug	DAPT with aspirin + clopidogrel 6w; SAPT with clopidogrel lifelong	FVIII level 100% for the procedure, ≥80% 3 days ≥30% on DAPT	No periprocedural complications; No thrombotic or coronary events and no bleeding complications at 9-month follow-up
Bhatti et al., 2019 [94]	60 y, F	HB, mild (15%)	3/NR	Sick sinus syndrome status post pacemaker implantation, TIA	Watchman PVI	VKA for 1 mo	FVIII level ≥ 30% on VKA	No periprocedural complications; No complications or FA at 6-month follow-up
Güray et al., 2019 [95]	67 y, M	HA, (baseline FVIII activity level ~10% with rFVIII)	3/3	Hypertension, HF	Amplatzer Amulet	DAPT with aspirin + clopidogrel 1 mo; SAPT with aspirin 2 mo	Adequate FVIII prophylaxis	No complications at 1-year follow-up
Coppola et al., 2020 [96]	Elderly, M	HA, severe (˂1%)	3/NR	Advanced arthropathy	Amplatzer Plug	SAPT with clopidogrel	FVIII level ≥80% during and 12 h after the procedure; >5% on SAPT	Clopidogrel stopped after 2 mo due to severe epistaxis and joint bleeds
	Elderly, M	HA, severe (˂1%)	3/NR	Advanced arthropathy	Amplatzer Plug	SAPT with clopidogrel	FVIII level ≥80% during and 12 h after the procedure; >5% on SAPT	NR
Toselli et al., 2020 [67]	76 y, M	HA, severe (˂1%)	3/3	Hypertension	Amplatzer Amulet	SAPT with clopidogrel 3 mo	FVIII level ˃60% before the procedure	Minor hemarthrosis and epistaxis while on SAPT No complications at 20-month follow-up
	73 y, M	HB, moderate	4/3	Cardiac bypass surgery, HF (LVEF 40%), hip replacement surgery	Amplatzer Amulet	DAPT 3 mo SAPT lifelong	FIX before the procedure	No complications at 12-month follow-up
	79 y, M	HA, severe (˂1%)	5/6	TIA, recurrent spontaneous hemarthroses, chronic kidney insufficiency, HCV-related chronic liver disease, treated hepatocellular carcinoma	Amplatzer Amulet	DAPT 3 wk SAPT 3 mo	FVIII level 65% before the procedure	Postprocedural acute pericarditis and mild transitory acute renal injury
Santoro et al., 2021 [97]	69 y, M	HB, moderate (3.5%)	3/3	DES for ACS, hypertension, melena and severe anemia while on DAPT and epistaxis while on SAPT— the patient refused FIX prophylaxis, hyperhomocysteinemia, curative treatment of low-grade transitional cell carcinoma, surgery for basal cell carcinoma	Left atrial appendage closure and cardioversion	Apixaban 2.5 mg bd, 1 mo DAPT with aspirin + clopidogrel 3 mo SAPT with clopidogrel lifelong	Eftrenonacog alfa	No postprocedural complications; No complications at 18-month follow-up
Lim et al., 2021 [54]	79 y, M	HA, mild (9%)	7/5	TIA, hypertension, PCI, atrioventricular node ablation and cardiac resynchronization therapy pacemaker, atherosclerotic calcifications at the carotid bifurcation and bulbs, HF (LVEF = 38%)	Watchman	VKA 6 wk SAPT with aspirin lifelong	FVIII level 100% for the procedure and 30% on VKA	No complications at 15-month follow-up
Dognin et al., 2021 [98]	61 y, M	HA, severe	2/1	NR	Watchman	No antithrombotic therapy	FVIII replacement	NR
Kramer, et al., 2021 [52]	70 y, F	HA, mild (14%)	5/3	Obesity, hypertension, HF	Amplatzer Amulet	SAPT with aspirin 6 mo	FVIII level ˃100% for the procedure; FVIII replacement 1–4 days after the procedure	Periprocedural arterial puncture
	75 y, M	HA, mild (20%)	2/2	Hypertension	Amplatzer Amulet	SAPT with aspirin 6 mo		Minor access-site hematoma and bleeding
	76 y, M	HA, mild (21%)	3/3	Hypertension	Amplatzer Amulet	SAPT with aspirin 5 mo		Self-limiting pericardial effusion Aspirin stopped due to major GI bleeding
	65 y, M	HA, mild (38%)	2/2	Hypertension	Watchman	SAPT with aspirin 6 mo		No complication
	60 y, M	HA, moderate (4%)	1/1	HF	Watchman	SAPT with aspirin 6 mo		Minor access-site hematoma and bleeding
	74 y, M	HA, mild (30%)	3/2	Hypertension	Watchman	DAPT 3 mo SAPT with aspirin 9 mo		Minor access-site hematoma and bleeding
	78 y, M	HA, severe (˂1%)	6/3	Stroke, recent AMI, DES implantation (2 mo previously)	Watchman	DAPT 1 mo SAPT with aspirin lifelong		Significant access-site bleeding Exitus 9 mo after LAAO due to staphylococcal sepsis and mitral valve endocarditis

HA = hemophilia A, HB = hemophilia B, VKA = vitamin K antagonist, SAPT = single antiplatelet therapy, DAPT = dual antiplatelet therapy, PVI = pulmonary vein isolation, FVIII = coagulation factor VIII, FIX = coagulation factor IX, NYHA = New York Heart Association, TIA = transient ischemic attack, LVEF = left ventricular ejection fraction, HCV = hepatitis C virus, DES = drug-eluting stent, ACS = acute coronary syndrome, PCI = percutaneous coronary intervention, AMI = acute myocardial infarction, HF = heart failure, LAAO = left atrial appendage occlusion, COPD = chronic obstructive pulmonary disease, CABG = coronary artery bypass grafting, ICA = internal carotid artery, GI= gastrointestinal; wk = week, mo = month, NR = not reported.

**Table 2:** Practical guide for long-term antithrombotic treatment.

Parameter	Recommendation
1. Patient characteristics	
The risk of stroke and systemic embolism	The CHA_2_DS_2_-VASc risk score assessment If 0, long-term ACO treatment should not be offered. If ≥1, long-term ACO treatment is recommended.
The bleeding risk	The HAS-BLED risk score assessment An attempt will be made to reduce the risk by intervening on modifiable factors.
	Assessment of the severity of hemophilia Coagulation factor level Coagulation factor replacement therapy Mandatory levels: ≥20% while on ACO ≥5–10% while on DAPT, SAPT
	The presence of inhibitors is a contraindication for ACO
2. Therapeutic intervention	
Rate control	The ACO indication is based on CHA_2_DS_2_-VASc risk score
Ablation	2 mo ACO, then the ACO indication is based on CHA_2_DS_2_-VASc risk score
LAAO	DAPT 1–6 mo, then lifelong SAPT
3. The anticoagulant treatment	
Type	NOAC preferred over VKA in HA patients NOAC preferred in HB patients
Dose	Low dose NOAC Apixaban 2.5 mg bid Dabigatran 110 mg bid Edoxaban 30 mg od Rivaroxaban 10 mg od VKA for INR 2–3
4. Patient preferences	
The patient should be informed of the advantages and disadvantages of the proposed treatments.	

ACO = anticoagulant, DAPT = dual antiplatelet therapy, SAPT = single antiplatelet therapy, mo = month, LAAO = left atrial appendage occlusion, NOAC = non-vitamin K oral anticoagulant, VKA = vitamin K antagonist, HA = hemophilia A, HB = hemophilia B.
